# An EXploration of the facilitators and barriers to paramedics’ assessment and treatment of pain in PAediatric patients following Trauma (EX-PAT)

**DOI:** 10.29045/14784726.2021.9.6.2.10

**Published:** 2021-09-01

**Authors:** Barry Handyside, Helen Pocock, Charles D. Deakin, Isabel Rodriguez-Bachiller

**Affiliations:** South Central Ambulance Service NHS Foundation Trust ORCID iD: https://orcid.org/0000-0002-1842-6582; South Central Ambulance Service NHS Foundation Trust ORCID iD: https://orcid.org/0000-0001-7648-5313; South Central Ambulance Service NHS Foundation Trust ORCID iD: https://orcid.org/0000-0002-2565-9771; South Central Ambulance Service NHS Foundation Trust ORCID iD: https://orcid.org/0000-0002-8526-064X

**Keywords:** ambulance, children, emergency medical services, pain, trauma

## Abstract

**Background:**

Pain is a common symptom among patients presenting to ambulance services and is often associated with traumatic injury. Assessment and management of pain in children in the pre-hospital setting is suboptimal. This study aimed to understand the facilitators and barriers experienced by paramedics in their assessment and management of pain in children who have sustained traumatic injuries.

**Methods:**

Face-to-face, audio-recorded semi-structured interviews using a piloted topic guide were conducted with paramedics employed by South Central Ambulance Service NHS Foundation Trust. Interviews were professionally transcribed, coded manually and analysed using thematic analysis.

**Results:**

Eleven interviews were conducted; three themes related to assessment and three related to management were identified. Previous positive experiences of utilising pain scoring tools were identified as a facilitator to pain assessment, whereas a lack of confidence in using pain scoring tools was a barrier. Patients’ understanding of and compliance with the tools were both a facilitator and a barrier to assessment. Facilitators to management included personal sub-themes of colleagues/others, exposure, being a parent, technology, severity of the injury and subjective pain scoring. Organisational facilitators included medicines, routes, and alternative methods. Situational facilitators included patient-specific solutions and parents. Five personal barriers to management included medicines, skill, consequences to self or patient, negative interactions, and limited exposure. Three organisational barriers included medicines and routes, equipment issues and choices, and training and culture. Within the theme of situation, two sub-themes emerged: patient-specific issues and environment-specific issues. Novel facilitators to emerge were those of alternative methods and being a parent.

**Conclusion:**

A multitude of factors incorporating situational, organisational, and personal all combine to determine how paramedics treat paediatric trauma patients. A multi-stakeholder approach to providing clearer assessment tools, improved education, equipment, and pharmacy options may improve assessment and management compliance for the benefit of the patient.

## Introduction

Pain experienced by paediatric patients is a common cause of presentation to ambulance services (JRCALC, 2019), trauma being one such reason. Whether the cause of pain is obvious or not, early analgesia for children is recommended (JRCALC, 2019) to negate short- and long-term physical and psychological consequences (Weisman et al., 1998; Young, 2005). Despite this, pain management of traumatically injured children is shown to be suboptimal in pre-hospital settings internationally and in the UK (Izsak et al., 2008; Lord et al., 2016; Pilbery et al., 2019; Swor et al., 2005; Whitley & Bath-Hextall, 2017).

Pain management and the underuse of analgesics in the pre-hospital setting (Swor et al., 2005) may be attributable to a deficiency in obtaining accurate pain scores, especially in children (Hennes et al., 2005; Murphy et al., 2014), among a number of other factors (Murphy et al., 2014; Walsh et al., 2013; Williams et al., 2012). JRCALC (2019) calls for children in pain to have their pain assessed for severity but paramedics have identified a number of difficulties with conducting this (Browne et al., 2016; Lerner et al., 2014; Lord et al., 2016 and Murphy et al., 2014). Despite a positive correlation between pain assessment and pain management appearing tenuous (Jennings et al., 2015, Lord et al., 2016; Pilbery et al., 2019; Whitley et al., 2020a), pain assessment has been shown to improve analgesic administration, albeit in an emergency department setting (Silka et al., 2004).

No qualitative study specifically focused on paramedic assessment and management of children in pain following trauma has been conducted before in the UK. International studies may not be representative of practice in the UK. This study sought to contextualise previous international findings by creating an in-depth understanding of current paramedic practice in the UK. Results will aid policy makers, guideline producers and UK ambulance services’ development and review of policies, protocols and educational provision around trauma pain assessment and management in children.

## Methods

### Aims

We sought to identify facilitators and barriers to the assessment and management of paediatrics, defined legally in the UK and in JRCALC (2019) as those between birth and up to 18 years old, in pain following acute traumatic injury. Acute traumatic pain was deemed any pain without a medical cause in the opinion of the paramedics interviewed.

### Design

This study employed an interpretative phenomenological approach within an interpretative paradigm, to gain an understanding of participants’ lived experiences (Holloway and Galvin, 2017). This strategy allows for a hermeneutic approach to be adopted, meaning the subjective nature of the data can be interpreted while acknowledging that the interviewer (BH) due to his background and employment cannot be truly detached from his assumptions and their effects on the data analysis (reflexivity) (Gill, 2020; Holloway and Galvin, 2017; Shaw, 2010).

In-depth semi-structured interviews were conducted because they are useful in the gathering of opinions, attitudes, and personal experiences (Wilson, 2014). The topic guide (see Supplementary 1) consisted of 17 questions developed from previous studies and in conjunction with patient and public involvement events (Murphy et al., 2014; Walsh et al., 2013; Williams et al., 2012). It was piloted among paramedics to ensure face and content validity, clinical relevance, and clarity. Interviews were conducted by BH, a novice interviewer but experienced paramedic known to some participants through his clinical, research and educator roles. This emphasises the importance of exploring reflexivity during the data analysis by BH and IR-B due to their clinical roles potentially influencing the analysis and results (Shaw, 2010). By doing so this improves ethical practice and sensitivity while reducing bias (Holloway and Galvin, 2017). BH’s multiple roles gave him greater credibility and understanding of the participants’ world and the language used within it, allowing for a more relaxed, open and informative interview process to capture more informative data and reduction in socially desirable answers (Holloway and Galvin, 2017).

Interviews took place at an ambulance station on a date and time convenient to each participant. A participant information sheet was provided before written consent was sought. The participant information sheet had reference to the Trust’s employee assistance programme, an anonymous service provided to all Trust staff to contact for advice and/or support for any issues they might experience either personally and/or professionally.

It was originally planned to conduct 12 interviews, but data collection finished at the point of *thematic saturation*, in the authors’ opinion when no new information was being produced. Interviews were not timed and could proceed for as long as necessary to answer all questions, probes, and the investigation of any other pertinent factors. Despite Hennink, Kaiser and Marconi (2017) questioning the rigour involved in thematic saturation, they identified that smaller numbers of interviews can produce comprehensive results especially in studies attempting to capture themes within a homogeneous population producing thick data.

### Setting

The study was conducted in the South Central Ambulance Service NHS Foundation Trust (SCAS). SCAS is a mid-sized UK ambulance service providing 999 emergency and urgent healthcare services across four counties: Oxfordshire, Berkshire, Buckinghamshire, and Hampshire. SCAS covers an area of 3554 square miles, with a population of >4 million. In 2019, SCAS received 626,153 999 calls. Of these, 96,380 (15.3%) were for paediatric patients of all causes. Of those calls for paediatric patients, 82,664 (85.7%) received a physical response, with 49,620 (60%) relating to a traumatic cause.

JRCALC (Joint Royal Colleges Ambulance Liaison Committee) provides clinical practice guidelines for all UK ambulance Trusts. However, there are regional variations based on service policy and protocol, training, equipment, and the use of Patient Group Directives (PGDs).

A variety of pharmacological and administration route options are available to SCAS paramedics. Nitrous oxide (entonox) is available as a self-administered inhaled gas for those compliant enough to self-administer. Oral analgesics include paracetamol (3 months +), ibuprofen (3 months +) (both liquid and tablet form) and oral morphine (12 months +). Parenteral options include intravenous (IV), intraosseous (IO), intramuscular (IM) and subcutaneous morphine (all 12 months +) and IV paracetamol (birth +) (via a PGD).

### Participants

Paramedics registered with the Health and Care Professions Council (HCPC) and currently employed by SCAS were invited to take part via a poster distributed through an internal staff publication and internal social media platforms. Word of mouth also took place. Purposive sampling was planned to be conducted but those who volunteered up to thematic saturation provided the required diverse sample. One volunteer expressed interest after data collection had finished while two others chose not to proceed after initial contact.

### Data collection and analysis

Interviews were audio recorded and transcribed verbatim professionally. All data was handled and transferred in respect of the Data Protection Act 2018 and local policy.

Using an inductive approach, BH and IR-B performed manual independent thematic analysis of data before collaborative analysis took place. An open-coding technique was used in accordance with Braun and Clarke’s (2006) six-step framework.

Once data analysis was finalised, member checking occurred to obtain feedback and to ensure the accuracy and completeness of results. SRQR reporting guidelines (O’Brien et al., 2014) were utilised to ensure the comprehensiveness of this qualitative study.

## Results

Eleven interviews were conducted up to the point of thematic saturation. Participant characteristics are presented in Table 1.

**Table 1.** Participant characteristics.

Participant number	Sex	Length of paramedic registration (median = 8 years)	Qualification achieved to obtain paramedic registration
P1	F	3 years	Foundation degree (FdSc)
P2	M	14 years	Institute of Healthcare and Development (IHCD) paramedic award
P3	M	2 years	Diploma of Higher Education
P4	M	13 years	IHCD
P5	F	12 years	IHCD (currently undertaking BSc)
P6	M	2 years	Bachelor of Science degree (BSc)
P7	M	2 years	Certificate of Higher education (CertHE)
P8	F	1 year	BSc
P9	F	9 years	FdSc (currently undertaking BSc)
P10	M	3 years	CertHE
P11	M	8 years	FdSc

Four domains were identified – barriers to pain assessment, facilitators to pain assessment, barriers to pain management and facilitators to pain management – with several themes and sub-themes creating these domains.

### Facilitators and barriers to pain assessment

The themes to emerge under these domains were very similar. *Patient’s understanding and compliance with pain assessment tools* was both a barrier and a facilitator to pain assessment. Some tools cannot be used by younger children:

From my experience they’re not really understanding … They’re just picking a number. (P7)

Other tools are more helpful in obtaining an accurate observation:

If they are older, I get them to point on the picture … (P2)

Where paramedics had previous positive experience(s) of using pain assessment tools, they gained confidence in this tool:

Where you’ve got the different faces … that has probably been the best thing we’ve had. I think it’s relatively accurate … most children you see will fit into that scoring system pretty well. (P2)

This was countered by a *lack of confidence in pain scoring tools*, implying they are not always thought of highly for use with this patient group, with a reliance on personal judgement of the pain:

But, yes, it’s so difficult. I don’t think we’ve got the scale right but what else have we got, use what we’ve got? (P10)

I don’t use them. I don’t use the smiley faces or anything like that. I just would go on experience, or I just describe things differently. (P5)

### Barriers to management

Within this domain three themes emerged, each of which comprised sub-themes (Table 2). Within the *personal* theme were sub-themes encompassing medicines, skill, consequences to self or patient, negative interactions, and limited exposure.

**Table 2.** Management domains, themes, and sub-themes.

Facilitators to pain management (themes and sub-themes)	Barriers to pain management (themes and sub-themes)
**Personal**	
Colleagues/others Exposure Being a parent Technology Severity of the injury Subjective pain scoring	Negative interactions Limited exposure Consequences to self or patient Skill Medicines
**Organisational**	
Medicines and routes Alternative methods	Equipment issues and choices Medicines and routes Training and culture
**Situational**	
Patient-specific solutions Parents	Patient-specific issues Environment-specific issues

Despite all participants referencing their JRCALC guidelines / PGDs before drug administration, there still appears to be a fear in the administration of certain medicines to this group of patients:

I think some people are pretty reticent or they don’t want to give children morphine, opiates, for some reason. I don’t know. People will – oh just give them Calpol or ibuprofen that’ll be alright or some paracetamol that’ll be good enough. (P10)

Several participants spoke about their reticence and/or lack of confidence in the skill of obtaining IV access on children:

I am reluctant to go IV on a child. I think that’s a confidence thing. (P5)

Consequences to self or patient was born out of a fear of something going wrong meaning an increased medical problem for the patient and subsequent disciplinary action against oneself:

… see it as a heavy-handed and strong pain relief and they’re scared that they’re then going to have side effects of that, they’re going to see drop off resps and level of consciousness and stuff … I think people think they’re going to have a child that’s not breathing on their hands. (P5)

Straight to HCPC jail. I think people in this job tend to fear the worst … There’s a lot of fear that if you make any clinical mistake, its straight to a hearing, many terrible things will happen. (P6)

Negative interactions with colleagues and/or other healthcare professionals have made paramedics doubt their abilities and what is best to do:

Yeah. I’d say it was negative kind of questioning of, well I wouldn’t have done that, why have you done that? Which does – I suppose it does kind of shape how you further treat people further down the line because you’re like – you regress on that experience and go, oh maybe I shouldn’t have done that, I’ll do something different. (P7)

Overall, there was widespread acknowledgement that several of these issues could stem from limited exposure to this category of patients:

I think there is limited experience and knowledge and exposure with paeds in trauma that I think, on a whole, we don’t do enough for them. (P5)

*Organisational barriers* included the sub-themes medicines and routes, equipment issues and choices, and training and culture.

Participants felt that the range of medicines they could administer and/or the routes of delivery to the patient were inadequate:

something which is more user friendly, easily accessible and potentially less side effects. (P11)

One participant expressed doubts that expanding the range would solve the problem, instead relating the issue to paramedic confidence:

So, I think there are other options out there that we could use. But then again if we brought them in would all the paramedics be confident in using them if they’re not confident in using opiates at present or they’re pretty reticent at doing that? (P10)

There was widespread call for more specific paediatric kit because of issues adapting adult ambulance issue equipment:

It’s the ones where you’ve got upper arm fractures. You’re trying to find the right size and their little arms are this big and you think I have nothing to fit that. (P8)

To assist with a holistic approach to the treatment of pain, a variety of other non-pharmacological options were offered to assist and make the situation more patient friendly:

numbing lotion. (P2)

I wish we carried stickers. (P5)

We could go for intranasal. (P8/10)

puzzle books. (P8)

Answers relating to the belief that paramedics do not receive adequate training in managing paediatrics and paediatric trauma were common. This was stated from both an employer and university perspective:

I think we probably need more training on paediatrics and how we deal with them in traumatic situations … (P7)

… in university not a lot got spent on any particular subject as a whole. It was very much a whistle stop tour around this is what being a paramedic is; good luck. (P8)

Within the theme of *situation*, relating to the incident itself two sub-themes emerged, patient-specific issues and environment-specific issues.

Patient-specific issues incorporated things such as age, fear, communication barriers and past medical history:

It’s trying to explain something to a child that’s not too alien. (P5)

Yeah, he definitely needed something but I no way could justify it because I had no idea about his history. (P4)

Environment-specific issues related to short distances to hospital; some participants stated a preference to defer pain relief to hospital staff:

If you’re literally two minutes away, would it not be easier just to deal with it for two minutes and then almost let the hospital do it when they’ve got a bit more practice. (P8)

The negative effect of parents’ presence on a clinician’s ability to provide pain relief was mentioned:

Yeah, a lot of the time you are treating the parents as well as treating the child. (P5)

And being stressed or being at the limit of one’s capacity with their current incident:

… those situations are normally more stressful than with an adult. (P3)

### Facilitators to management

The same three themes that were identified as barriers were also identified as facilitators: personal, organisational, and situational.

The *personal* theme was made up of sub-themes of colleagues/others, exposure, being a parent, technology, severity of the injury and subjective pain scoring.

When there were good levels of support from colleagues/others (especially healthcare professionals), this seemed to relax paramedics and remove some of their personal blocks to adequate treatment practices:

I was backed up by an experienced technician crew, which was really helpful … so he was able to take over that Entonox coaching and just chatting to the lad. (P2)

The more experienced paramedics were happy to have had exposure to similar cases to draw lessons from, believing the more incidents you attend of this nature the more experience you have to draw on to assist in your decision making:

I think this is where experience plays a big part and where you’ve seen things previously. (P5)

Those who were parents were adamant that this assisted with their treatment regimes. They alluded to the experience it brought in terms of communicating with children, understanding developmental stages, being aware of children’s lifeworlds and knowing how difficult it can be to administer medicines to children and therefore being more assertive:

I think for me, having got – being a mum and having children, I manage children differently now than what I would have done before, just having been there and knowing … manage children more effectively once you’ve got children of your own. (P5)

Technology was mentioned to be assistive at times when history gathering was difficult. This is likely to have an increasing effect on paramedic abilities in the future:

Well these days we have access to patient records through the Smartcard so I would look at that and see if there was anything on there. (P4)

Every participant referred to utilising their own personal perception of the pain based on the severity of the injury as motivation to treat pain more aggressively:

I think it would be the severity of the incident, severity of the trauma. That would go for adult or child. If you’re going to anyone who has got multiple injuries and appearing to be in excruciating pain, you don’t need to ask some patients if they’ve got a 10 out of 10 pain, that you can see it. (P5)

So, I guess you map your analgesia to the level of what you see in front of you, whether that’s the overt things like the injury or whether that’s the way the child is reacting. (P2)

Several participants mentioned that because of the subjectivity of pain scoring tools they would supersede this with their own clinical judgement of the pain and treat accordingly:

I think as a paramedic you can tell by looking at someone can’t you if they’re really in pain or if they’re just sat there gazing around kind of thing. I think I would take my impression into account with the pain score … if they were showing signs that they were in pain, then I would do it. (P9)

… but for such a significant injury they seemed quite happy with it, so I suppose my treatment may have reflected my perception of the pain that they should be in, as opposed to the pain they were displaying, because I was quite conscious I didn’t want to undertreat what should have been a quite significant painful injury. (P6)

*Organisational* facilitators included the sub-themes of medicines and routes, and alternative methods.

The range of medicines and routes was deemed adequate by many of the participants considering the broad spectrum they cover:

I think we’re fortunate that we have several medications that we can give to children that go right from the bottom end with paracetamol and ibuprofen, right the way to morphine at the top. (P2)

Alternative methods, including physical (splinting, cooling etc.) and psychological (distraction, parents, toys etc.) methods of pain relief, were universally described as effective:

I would say, hand on heart, there has never been a traumatically injured paediatric patient where I’ve not done anything for their pain, even if its splinting. (P2)

So, I very much use and utilise what’s around me to a) sort of get their reassurance and get them onside and know that I’m not trying to do anything to hurt them anymore or to scare them. (P5)

Within the *situational* domain were sub-themes of patient-specific solutions and parents.

Patient-specific solutions were based on a child’s ability to understand and comply, making it easier to manage pain:

… I think just because of her compliance really and her understanding levels at that age was quite good …. (P10)

Several participants mentioned the need to treat a child differently, suggesting that ensuring paediatric comfort is a greater priority for them than for adults:

I think maybe most people would – don’t like to see a child in pain certainly not so maybe you would – … Maybe you would go in more heavy handed than you would light handed if it was an adult. Would you let an adult sit in moderate pain rather than a child in moderate pain or would you take that little bit more of a temper on it … (P10)

The second sub-theme of parents pertains to the joint decision-making regarding treatment that involves parents and the calming, supportive effect they can have:

So, analgesia wise we discussed it with parents, we discussed it with her and she was happy that she could take Oromorph and paracetamol just for that age range of that child. (P10)

## Discussion

This is the first qualitative study based in the UK exploring paramedics’ barriers and facilitators to the assessment and management of traumatic pain in children who present to the ambulance service.

All barriers identified in this study have been previously discovered (Browne et al., 2016; Hennes et al., 2005; Holmström et al., 2019; Murphy et al., 2014; Rahman et al., 2015; Walsh et al., 2013; Whitley et al., 2017; Whitley et al. 2020b; Williams et al., 2012).

Common facilitators identified include the availability of assistive guides, (Williams et al., 2012), increased age (Browne et al., 2016; Watkins, 2006), parental involvement (Whitley et al., 2020b; Williams et al., 2012), positive relationships with other healthcare professionals (Whitley et al., 2017; Whitley et al. 2020b; Williams et al., 2012), limited exposure (Murphy et al., 2014), range of medicines and delivery methods (Murphy et al., 2014) and personal perception of pain (Murphy et al., 2014; Rahman et al., 2015; Walsh et al., 2013). Previously unrecognised facilitators include the use of alternative pain relief methods by physical and/or psychological methods rather than utilising pharmacological analgesia and being a parent.

The novel facilitator of being a parent, though seen as a facilitator here due to the psychosocial benefits this may bring the patient, could potentially also be a barrier. A clinician becoming too emotionally involved or conversely too stoic may cloud their decision making, leading to reduced care. Exploring the dynamic and effect of clinicians who are parents and the subsequent care provided compared to by non-parents would be a worthy area for future research, as agreed by Whitley et al. (2020a).

This facilitator may help to explain the other novel facilitator of using alternative methods, but Whitley et al. (2020a) did not find these methods made a significant difference. However, reduction in pain without pharmacological intervention has been reported, with alternative methods given as a possible reason (Jennings et al., 2015; Lord et al., 2016; Pilbery et al., 2019). Despite the general agreement of their effectiveness, there was a call for improved paediatric-specific equipment. These methods extend beyond the physical and involve play, distraction and reducing fear and anxiety. Clinicians who are comfortable around children may create better environments and rapport with their patient(s), helping to reduce the anxiety of the child and subsequently of their parents. This study has reported that parents can prove to be facilitators and barriers, as did Whitley et al. (2020b). Understanding the dynamics between patient, parent and clinician could be useful in reducing barriers to assessment and management.

The most commonly recorded facilitators were based on paramedics’ personal perception of pain relating to the severity of the injury and assessing pain via subjective means (as also found by Jones & Machen, 2003; Murphy et al., 2014; Walsh et al., 2013; Whitley et al., 2020b). This is quantified by Rahman et al. (2015), who showed that paramedics were six times more likely to use clinical judgement over pain scoring tools in children than in adults for opioid administration, and by Whitley et al. (2020b), who reported traumatic pain as more readily treated. JRCALC (2019) does advocate for clinical decision making to supersede the stepwise approach to analgesia if appropriate. Caution is advised because large underestimations of pain can occur (JRCALC, 2019; Lord & Woollard, 2011); hence the call for pain scores to be the fifth vital sign (American Pain Society Quality of Care Committee, 1995) to improve accuracy. Despite their limitations, pain scores should be recorded both pre- and post treatment using tools validated within paediatric emergency departments (JRCALC, 2019; Whitley & Bath-Hextall, 2017; Whitley et al., 2020b). This study supports the recommendation of a concerted effort to develop a reliable and valid tool for use by pre-hospital clinicians (Hennes et al., 2005).

Paramedics go through complex decision-making processes (Jones & Machen, 2003). Negative interactions with other healthcare professionals (HCPs) can damage confidence in decision making (Murphy et al., 2014; Williams et al., 2012) but positive interactions/relationships can build confidence (Whitley et al., 2017; Williams et al., 2012). Murphy et al. (2014) advocate the development of a multi-stakeholder protocol to assist decision making and limit the disjointed expectations between acute and primary care.

Lack of exposure, also seen by Rahman et al. (2015), Whitley et al. (2020b) and Williams et al. (2012), and lack of education, also reported by Murphy et al. (2014), were commonly mentioned. Universities and ambulance Trusts could explore ways to improve exposure and clinical development opportunities regarding paediatric patients, helping to create empowered, knowledgeable, and skilful clinicians. eLearning and simulation have been advised (Murphy et al., 2014).

Further barriers related to medicines, skill application and subsequent consequences to the patient have all been previously reported (Hennes et al., 2005; Murphy et al., 2014; Whitley et al., 2017; Williams et al., 2012). There was widespread fear in cannulating children, despite Browne et al. (2016) showing that more opioids are administered if successful. A different method to IV administration suggested was fentanyl for Intranasal (IN) administration, reported by Whitley et al. (2020b) as a facilitator and as being safe and effective in paediatric patients in pre-hospital settings (Setlur & Friedland, 2018). It was mentioned that this may not solve paramedics’ reticence in providing analgesia, which was also alluded to by Whitley et al. (2017) when discussing that new medicines do not always mean improved rates of administration by paramedics.

A desire to alleviate pain due to the patient being a child was a sub-theme facilitator. Albeit that this is an encouraging finding, the previous literature does highlight that this might not always translate into practice (Browne et al., 2016; Hennes et al., 2005; Izsak et al., 2008; Lerner et al., 2014; Lord et al., 2016; Pilbery et al., 2019; Swor et al., 2005; Whitley & Bath-Hextall, 2017). Whether this means a preference to treat with more readily available medicines and to defer stronger analgesic drugs due to the numerous barriers present would need further investigation. What it highlights is the obvious discordance within the profession between paramedics’ intentions (based on knowledge, skills and confidence) and their subsequent actions.

## Limitations

The interview protocol, while internally piloted, was not externally validated. Despite reassurances of anonymity, participants may have felt obliged to provide socially desirable responses due to the interviewer being a colleague and clinical education manager.

No absolute saturation strategy was employed; it was at the discretion of BH as to when he believed no new data was being produced that data collection finished.

The interviews were transcribed by a third party due to resource constraints. This might have meant the researcher not being fully immersed in the data. Braun and Clarke’s (2006) six-step framework calls for familiarisation of the data before coding begins. Relistening to the audio recordings and reading the transcripts on at least two occasions occurred. An independent researcher also conducted results analysis before discussion and agreement of the results.

While this was a study looking at paramedics’ experiences, it did not include the experiences of children who were patients or their parents. By obtaining this data it could triangulate what has been found and present a different point of view.

## Conclusion

This study supports previously identified facilitators and barriers to the assessment and management of pain in children but also introduces some novel ones. There appears to be a disparity within the paramedic profession between what paramedics want to do and what they end up doing / are able to do. Despite paramedics’ best intentions, numerous factors appear to block optimal care, leading to suboptimal patient experience. Increased awareness of and exposure to assessment tools, guidelines, pharmacology, paediatric-specific equipment and skill practice utilising innovative education methods may overcome some of these barriers while also promoting and strengthening the revealed facilitators.

## Author contributions

BH: concept and study design, collection, analysis, and interpretation of the data and drafting of the work. HP: concept and study design, drafting of the work, critical revision, and approval of the final work. CD: concept and study design, drafting of the work, critical revision, and approval of the final work. IR-B: analysis and interpretation of the data and drafting of the work. CD acts as the guarantor for this article.

## Conflict of interest

All authors work for the Trust in question. The lead researcher was known to several of the participants.

## Ethics

Ethical approval was not required for this study, but SCAS’s Clinical Review Group gave permission for it to take place. The research was conducted according to the principles of the Declaration of Helsinki. Health Research Authority (HRA) approval was sought and gained for this study. IRAS project ID: 269082, Protocol number: PRO/001/2019/BH, REC reference: 19/HRA/5888.

## Funding

The College of Paramedics provided the funding for this research project.
